# Low-dose coronary artery calcium scoring compared to the standard protocol

**DOI:** 10.1007/s12350-022-03120-3

**Published:** 2022-10-26

**Authors:** Ileana Rosely Allio, Federico Caobelli, Cristina Elena Popescu, Philip Haaf, Ian Alberts, Simon M. Frey, Michael J. Zellweger

**Affiliations:** 1 Department of Cardiology, Clinic of Cardiology, University Hospital Basel, University of Basel, Petersgraben 4, 4031 Basel, Switzerland; 2 Clinic of Radiology and Nuclear Medicine, University Hospital Basel, University of Basel, Basel, Switzerland; 3 Clinic of Nuclear Medicine, Kantonsspital Baden, Baden, Switzerland; 4 University Clinic of Nuclear Medicine, University of Bern, Bern, Switzerland

**Keywords:** Coronary calcium score, myocardial perfusion imaging, low-dose CT, coronary CT, attenuation correction

## Abstract

**Background:**

We aimed to compare coronary artery calcium scoring (CACS) with computed tomography (CT) with 80 and 120 kVp in a large patient population and to establish whether there is a difference in risk classification between the two scores.

**Methods:**

Patients with suspected CAD undergoing MPS were included. All underwent standard CACS assessment with 120-kVp tube voltage and with 80 kVp. Two datasets (low-dose and standard) were generated and compared. Risk classes (0 to 25, 25 to 50, 50 to 75, 75 to 90, and > 90%) were recorded.

**Results:**

1511 patients were included (793 males, age 69 ± 9.1 years). There was a very good correlation between scores calculated with 120 and 80 kVp (*R* = 0.94, *R*^2^ = 0.88, *P* < .001), with Bland–Altman limits of agreement of − 563.5 to 871.9 and a bias of − 154.2. The proportion of patients assigned to the < 25% percentile class (*P* = .03) and with CACS = 0 differed between the two protocols (n = 264 vs 437, *P* < .001).

**Conclusion:**

In a large patient population, despite a good correlation between CACS calculated with standard and low-dose CT, there is a systematic underestimation of CACS with the low-dose protocol. This may have an impact especially on the prognostic value of the calcium score, and the established “power of zero” may no longer be warranted if CACS is assessed with low-dose CT.

**Supplementary Information:**

The online version contains supplementary material available at 10.1007/s12350-022-03120-3.

## Introduction

Coronary artery calcium score (CACS) is easily assessed noninvasively by cardiac computed tomography (CCT) and is a robust and well established tool for risk stratification of patients with suspected coronary artery disease (CAD).^1^ If performed along with a myocardial perfusion scintigraphy tomography (SPECT-CT) it provides complementary information for image interpretation and incremental prognostic value.^2^ Moreover, in a recent study a very low long-term mortality rate was reported in patients without coronary calcifications (CACS = 0), while higher CACS were associated with higher risk of cardiac events over a 15-year follow-up period.^3^

In clinical practice, CCT for the assessment of CACS is commonly acquired with a CT tube voltage set at 120 kilovolt peak (kVp), resulting in an effective radiation dose of approximately 0.6 mSv.^4^ In addition, a subsequent low-dose CT with 80 kVp tube voltage is commonly acquired for attenuation correction (AC) purpose.^5^

In order to address the requirement for population dose containment, the possibility to use a single CT scan with tube voltage set at 80 kVp both for AC and for the assessment of CACS would be highly warranted, as recently suggested in other studies featuring small patient cohorts.^4,6^ The preliminary findings of these latter studies need now to be validated in larger patient populations. The aim of the current study was twofold:

1. To compare CACS based on CT scan protocols of 80 and 120 kVp in a large population of consecutive patients.
2. To assess whether there is a difference in the assignment to the percentile classes^7,8^ between the two scores, especially in patients with CACS = 0.

## Materials and methods

### Study design

This single-center retrospective observational study was conducted in accordance with the declaration of Helsinki. A waiver for retrospective analysis of the patient’s data was obtained by Cantonal Ethics Commission. Consecutive patients without prior history of CAD undergoing myocardial perfusion scintigraphy (MPS) as part of standard clinical care were included.

All underwent standard CACS-CCT scanning with 120-kVp tube voltage and an additional CCT scan with 80 kVp, which was also used for attenuation correction (AC).

### Imaging protocol

All patients sequentially underwent a standard non-contrast enhanced CT scan (tube voltage 120 kVp, 25 mAs, pitch 1.2, rotation time 2.1 ms, Matrix 128 × 128, collimation 1.6 × 1.2) and a low-dose CT acquisition (tube voltage 80 kVp, 20 mAs, pitch 1.2, rotation time 0.6 ms, Matrix 128 × 128, collimation 1.6 × 1.2). Standard scans were performed in cranio-caudal direction during inspiratory breath-hold with prospective electrocardiogram (ECG)-triggering. Low-dose scans were performed also in cranio-caudal direction, but without breathing instruction and without electrocardiogram (ECG)-triggering. All post-acquisition processing and reconstruction were done in a similar fashion for both the low and standard dose scans. Radiation Dose was approximately 0.6 mSv for the standard protocol and 0.2 to 0.3 mSv for the low-dose protocol.

### Image Evaluation

All images were read in consensus by a board-certified nuclear medicine physician and a board-certified cardiologist.

Calcium scoring was done, equally for the 80 and 120 kVp acquisitions using a dedicated semiautomatic software included in Syngo.Via workstations (Siemens Healthineers AG, Erlangen, Germany). In brief, all pixels with an attenuation equal or above the lowest threshold (i.e., ≥ 130 HU) having an area ≥ 1 mm^2^ are automatically color-marked and then manually selected by creating a region of interest around all lesions found in a coronary artery. The software then calculates the CACS as previously reported,^9^ by multiplying the density score and the area of calcification.

Based on the CACS derived from both, the 80 and 120-kVp scans, each patient was allocated to the corresponding percentile group as reported in the literature^9^: < 25%, 25% to 50%, 50% to 75%, 75% to 90%, > 90%.

### Statistical analysis

Continuous variables are shown as mean with standard deviation unless otherwise specified, *P* values less than 0.05 were taken to indicate statistical significance. CACS were compared between scans with different tube voltage using Wilcoxon Test after confirmation of a non-normal distribution by means of Kolmogorov–Smirnov test. Statistical equivalence between 80 and 120-kVp CT scan was also assessed by calculating moment correlation coefficients (*R* and *R*^2^) and values were further analyzed by means of Bland–Altman Analysis.

Fisher Exact Test was used to compare differences in the assigned percentile class between 80 and 120-kVp scans. SPSS Software Version 22.0 for Windows was used.

## Results

### Patient demographics

The study population comprised 1511 consecutive patients (of whom 793 were male (52.5%), mean age 68 ± 9.1 years), all consecutively included between October 2016 and March 2018. The patients had a high cardio-vascular risk profile (BMI 29 ± 5.4 kg/m^2^, 35.6% with diabetes mellitus, 69% with arterial hypertension, 20.6% had a family predisposition, and 48.5% had dyslipidemia) as reported in Table 1. Prior to image acquisition all patients underwent adequate stress-testing, either pharmacological or with ergometry.

**Table 1:** Patients’ characteristics

Characteristic	Value
Age (range years)	68 (59–77)
Male	792 (52.5%)
BMI kg/m^2^	29 ± 5.4
Diabetes	539 (35.6%)
Family history of CAD	311 (20.6%)
Hypercholesterinemia	733 (48.5%)
Arterial hypertension	1043 (69.0%)
Smoking or prior history of smoking	572 (37.9%)

### CAC scoring

With regard to standard CT acquisitions with 120 kVp tube voltage, median CACS was 134 (inter-quartile range 8 to 625). 378 (25.0%) patients were assigned to the < 25% percentile class, of whom 264 (17.5%) had CACS = 0. Patients assigned to the other percentile classes were 259 (17.1%) in the 25% to 50%, 341 (22.6%) in the 50% to 75%, 252 (16.7%) in the 75% to 90%, and 281 (18.6%) in the > 90%, respectively.

Considering low-dose acquisitions with 80 kVp tube voltage, median CACS was 52 (inter-quartile range 0 to 379). 591 (39.1%) patients were assigned to the < 25% percentile class, of whom 437 (29%) had a CACS = 0. Patients assigned to the other percentile classes were 236 (15.5%) in the 25% to 50%, 297 (19.7%) in the 50% to 75%, 200 (13.3%) in the 75% to 90%, and 187 (12.4%) in the > 90%, respectively.

**Figure 1:**
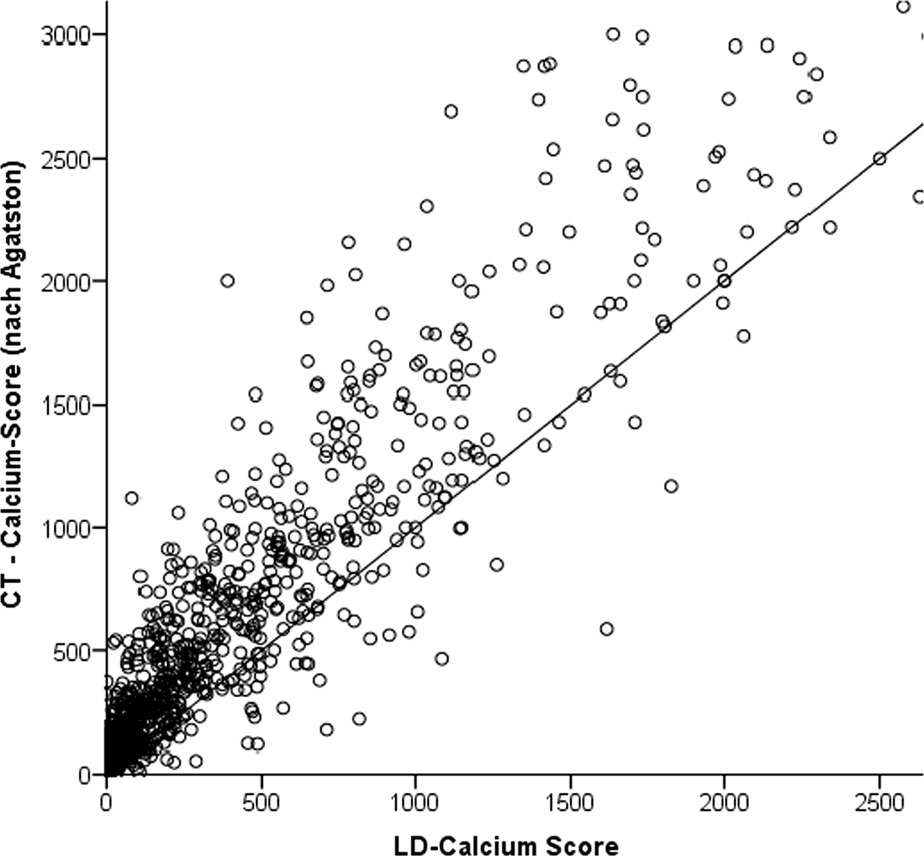
Correlation between CACS obtained with standard and very low-dose protocols

**Figure 2:**
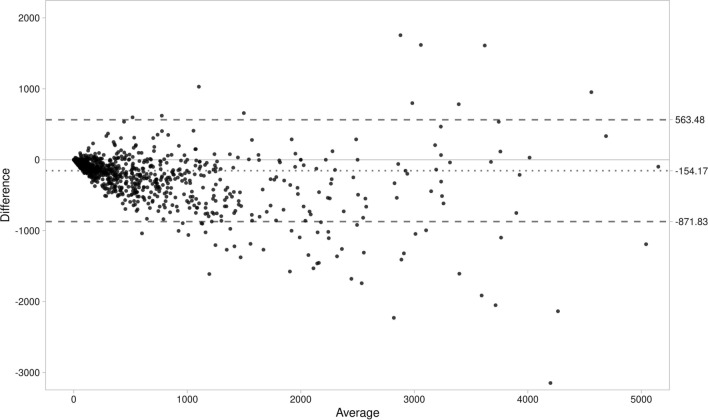
Bland–Altman analysis comparing CACS obtained with standard and low-dose protocol

Overall, there was a very good correlation between scores calculated with 120 and 80 kVp tube voltage scans (*R* = 0.94, *R*^2^ = 0.88, *P* > .001). Bland–Altman limits of agreement of − 563.5 to 871.9 and a bias of − 154.2 (Figures 1, 2).

**Figure 3:**
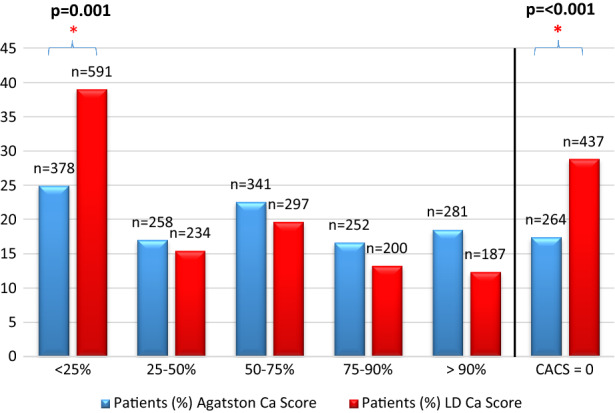
Proportion of patients in the different CACS classes. (*) indicates statistical significance

There was a difference between the proportion of patients assigned in the < 25% percentile class (*P* = .03) (25.0% in standard CT acquisition vs 39.1% in low-dose CT acquisition). Conversely, the proportion of patients assigned to the percentile classes other than < 25% did not differ between the two acquisition protocols. The results are summarized in Table 2 and in Figure 3.

**Table 2:** Assignment of the included patients in the relevant percentile class

Ca score percentile		Total	Men	Women	BMI > 25	BMI > 30
< 25%	Agatston	378 (25%)	153 (19.3%)	225 (31.3%)	271 (24.3%)	112 (22.2%)
	LD CACS	591 (39.1%)	269 (33.9%)	322 (44.8%)	427 (38.2%)	190 (37.6%)
	*P*	< .001	< .001	.001	< .001	< .001
	95% CI	0.744–0.886	0.729–0.919	0.704–0.917	1.25–1.47	1.25–1.59
Of whom	Agatston	264 (17.5%)	71 (9.0%)	193 (26.9%)	190 (17.0%)	89 (17.6%)
CACS = 0	LD CACS	437 (28.9%)	143 (18.0%)	294 (40.9%)	321 (28.7%)	155 (30.7%)
	*P*	< .001	.05	.001	< .001	< .001
	95% CI	0.794–0.934	0.810–1.001	0.711–0.918	1.25–1.48	1.23–1.57
25–50%	Agatston	258 (17.1%)	164 (20.7%)	94 (13.1%)		
	LD CACS	234 (15.5%)	143 (18.0%)	91 (12.7%)		
	*P*	.63	.55	.93		
	95% CI	0.943–1.102	0.926–1.153	0.899–1.123		
50–75%	Agatston	341 (22.6%)	206 (26.0%)	135 (18.8%)		
	LD CACS	297 (19.8%)	176 (22.3%)	121 (16.9%)		
	*P*	.81	.39	.68		
	95% CI	0.958–1.124	0.939–1.177	0.914–1.148		
75–90%	Agatston	252 (16.7%)	128 (16.2%)	124 (17.3%)		
	LD CACS	200 (13.2%)	103 (13.0%)	97 (13.5%)		
	*P*	.31	.50	.44		
	95% CI	0.964–1.125	0.933–1.154	0.934–1.170		
> 90%	Agatston	281 (18.6%)	141 (17.8%)	140 (19.5%)		
	LD CACS	187 (12.4%)	100 (12.9%)	87 (12.1%)		
	*P*	.06	.26	.13		
	95% CI	0.996–1.163	0.955–1.183	0.975–1.222		

There is a higher prevalence of patients assigned to the < 25% percentile class in the Low-dose (LD) CT scans

Moreover, we found a significant difference in the proportion of patients with CACS = 0 between standard acquisition (n = 264, 17.5%) and low-dose scan (n = 437, 28.9%, *P* < .001). The 437 patients with CACS = 0 with the low-dose scan had CACS ranging between 0 and 372 (median 0, IQR 0 to 4). Among patients with CACS = 0 in the low-dose imaging, 173 had CACS > 0 on the standard acquisition. Of note, these 173 patients are mostly coming from the < 25% risk class as assessed with the standard protocol. In only 9/173 Patients (5%) the standard acquisition showed markedly higher values (i.e., from the 25% to 50% risk class, median 6, IQR 1 to 3).

Among those patients assigned to the < 25% percentile class, the proportion of patients with BMI ≥ 25 and BMI ≥ 30 was higher for the low-dose protocol compared to the standard one (38.2% vs 24.3% and 37.6% vs 22.2%, respectively, both *P* < .001). The same held true for patients with CACS = 0 (28.7% vs 17.0% and 30.7% vs 17.6%, respectively, both *P* < .001). Of note, the difference in the proportion of patients assigned to the < 25% percentile class was independent of patient sex.

## Discussion

CACS is a useful prognostic tool to accurately estimate long-term risk of asymptomatic or symptomatic patients without prior CAD as well as for initial high-risk patients.^10,11^ It has been shown that patients without coronary calcifications of any degree have a very low mortality risk compared with the general population, while cardiovascular risk progressively increases with higher CAC scores.^12^

Therefore, the reliability of CACS results is pivotal in the prognostic assessment of patients with suspected CAD, and to identify those patients with the lowest risk of adverse events. Standard protocols for the assessment of CACS fit this need but cause a non-negligible additional radiation burden. It is therefore incumbent upon physicians performing cardiovascular imaging to reduce the radiation burden associated with CACS as far as is practicable. While the mean radiation burden associated with medical procedures has considerably decreased in recent years, there is still the potential for further reduction. An elegant way to reduce the radiation burden is to modify the parameters of CCT acquisition, provided that diagnostic accuracy can be maintained.^13^

Some studies demonstrated the accuracy of CACS acquired with low-dose protocols using tube voltage set at 80 to 100 kVp. A first phantom study showed an equivalence in accuracy and reproducibility using tube voltages set at 120 and 80 kVp.^14^ Hecht et al. demonstrated that using protocols with low-dose lung scanning allows for an excellent agreement of CACS-based risk classification at low and standard doses.^15^

Conversely, other two studies comparing 120 and 100 kVp showed that low tube voltages lead to a systematic overestimation of CACS.^16,17^ Consistent with these latest results, different HU thresholds were proposed to assess the CACS (e.g., 147 HU instead of 130 HU^16^).

In this regard, there is a discrepancy between the results of our study and those reported in the literature. In fact, we found a systematic underestimation of calculated CACS if 80 kVp tube voltage was used. As a consequence, the number of patients assigned to the low (< 25%) percentile class was different between the two protocols, being higher for low-dose acquisitions. Additionally, also the proportion of patients with CACS = 0 was different between the two acquisition protocols. The discrepancy may relate in a difference in the patient sample size, being ours larger by a factor of 10 compared with the previous reports. Another possible explanation is the impaired recognition of smaller calcifications with the low-dose protocol, possibly due to increased noise and consequent loss in spatial resolution. In a recent study, reducing the kVp tube voltage caused an increase in image noise by a factor 1.9 and 2.5 at 80 and 70  kVp, respectively.^18^ Due to image blurring and the concomitant impact of partial volume effect (PVE),^19^ it is conceivable that some tiny lesions are missed in low-dose protocols, and this becomes evident when larger patients’ samples are investigated.

It could be argued that increased noise at low dose may also cause overestimation in view of lesions which may be wrongly considered as calcium. Alternatively, this overestimation may come from an apparent increase in surface area of the detected calcifications. However, calcium scoring relies on two parameters: weighted density score given to the highest attenuation value and the area of the calcification. Artifact-related lesions might not present with sufficient density to be included in the automatic calcium scoring. On the other hand, even true calcification with increased apparent surface area may not lead to an overestimation of CACS; if images are blurred, density may appear lower. Even a relatively small difference in HU can cause a difference in the weighted density score, which may not be counterbalanced by the increase in area, especially if the density of detectable calcifications falls below 130HU.

In this regard, our results show that the greatest difference and spread in CACS between the two scores becomes more evident in the progressively higher scores and not in the very lowest percentile groups. This observation seems to rule out increased noise as major determinant of underestimated CACS, as the impact of noise should be more evident for small calcifications, as seen in lower CACS. However, as CACS equals the sum of scores of all calcifications, then higher CACS often results from multiple lesions. As a real calcification can be missed or underestimated if a blurred image causes a spread of its appearance, the same repeated error on multiple calcifications would amplify the bias in the calculation of global CACS with consequent underestimation, which intuitively becomes more evident if a large patient population is investigated.

Also, BMI can substantially affect imaging quality. It was shown that BMI is a major factor to predict image quality in patients undergoing a thorax CT: the higher the BMI, the lower the image resolution.^20^ This was also the case in our study, wherein the proportion of patients with BMI ≥ 25/30 was higher in those patients with lower CACS using our low-dose protocol. The fact that the calculation of CACS can be affected by high BMI, especially if a low-dose protocol is used, has evident implications in clinical practice and suggests that overweight and obese patients should be scanned with standard protocols in order to obtain a reliable calcium scoring. The impact of sex in this regard seems negligible, as the higher proportion of patients in the < 25% percentile class as well as with CACS = 0 was constant across male (*P* = .50) and female patients (*P* = .11).

In addition, the lack of breathing instruction in the low-dose protocol may also explain decreased resolution. In fact, it is well known that the patient’s motion can have a major impact on image quality on CT.^21^ In this regard, we may hypothesize that the relatively short acquisition time and the patients’ compliance could minimize the effect of the lack of breathing indication on the detectability and the precise assessment of small calcifications, but a definite answer cannot be given and should be investigated in future works. It should be noted that all patients were cooperative and that a low-dose CT during MPS for attenuation correction purpose only lasts for one single breath cycle, thus reducing the risk of major motion-related artifacts.

In our paper, we did not use the recently proposed HU thresholds for 80 kVp tube voltages and our choice deserves a further clarification. Gräni et al. proposed in 2018 adapted thresholds for acquisition with tube voltages down to 70 kVp,^6^ in view of a tendency in their study toward an overestimation of CACS if lower voltages were deployed. In our study the use of such threshold would have rather amplified the underestimation, with evident impact on diagnostic accuracy. Hence, we believe that the use of the same HU thresholds between the two protocols (i.e., 130 HU) is justified here. While the calculation of CACS may be affected by these drawbacks, the fact that the CACS calculated with the standard acquisition within the same patients differed only in a small proportion of patients and to a small extent renders the overall clinical impact probably minor with respect to the assignment to the established risk classes.

However, the impact on the diagnosis (normal vs abnormal) and on the prognostic value should be further discussed. In fact, if a low-dose CCT is used, significantly more patients are considered as not having any coronary calcifications, and this may lead to an underestimation of the cardiovascular risk in these patients. In our patients’ population, 173 Patients (11.6%) were falsely diagnosed as non-CAD due to CACS = 0 with the low-dose protocol, but > 0 with the standard protocol (median 6, IQR 1 to 3). Of note, all patients with CACS = 0 with the standard protocol also had CACS = 0 with low-dose protocol. A low calcium score is still consistent with a favorable prognosis, with the exception of very young patients, as the majority of events occur in individuals with high CACS percentile classes.^8^ As such, consistent with data from the literature, CACS percentile classes may constitute an effective screening method to stratify individuals at risk as well, and in this regard, our data showed a very good correlation across percentiles classes (*r* = 0.857, *r*^2^ = 0.74, *P* < .001). But as a matter of fact, the power of zero^22^ and its ability to predict a long disease-free survival in patients with suspected CAD^22^ should be considered reliable only if the CACS is assessed by standard protocols. This latter aspect has an evident impact in clinical practice, wherein patients are normally referred to calcium scoring as primary prevention if asymptomatic. In such patients, a precise diagnosis of CAD is essential to plan an adequate primary prevention. The same does not hold true for a patients’ population similar to that of the present study, wherein patients were referred to an ischemia test in view of their high cardiovascular risk and/or symptoms. While a standard protocol is mandatory if CACS is calculated as standalone modality, the same may not hold true if an ischemia test such as MPS is associated. In this latter setting, the implementation of low-dose protocols for the calculation of CACS may be pursued without relevant loss in prognostic power. Thus, low radiation dose protocols might be used without relevant impairment in accuracy, thus maintaining a reliable risk stratification for medical therapy.

Our study has some limitations. Due to the retrospective nature of the present study, the impact of technical artifacts potentially affecting image quality could not be assessed. It should be noted that standard parameters were used regardless of body size, sex and age and were not standardized between the two protocols. Hence, the impact of single parameters (e.g., mAs and rotation time) on calcium scoring could not be assessed as well. Furthermore, we could not investigate the lowest limit of dose reduction able to yield equivalent diagnostic accuracy. It is conceivable that a further reduction in tube voltage may be pursued while maintaining an adequate diagnostic performance, but this concept should be validated in further studies. We could not assess the prognostic value of our calculated CACS with both protocols as no complete follow-up data were available. Specifically, it still needs to be elucidated, whether the small calcifications which have been misdiagnosed with the low-dose protocol would have an impact on patients’ prognosis or on the ability to predict cardiac events. As such more work is needed to clarify whether the same prognostic value of CACS as calculated on standard protocols also pertains to low-dose protocols, and the incremental value over MPS alone should be elucidated. While this was beyond the scope of the present paper, this is of importance to evaluate the real impact on clinical practice. Finally, as mentioned before, the HU thresholds used in this study were the same for standard and low-dose acquisitions. Whether a different adaptation of thresholds is necessary, for example in the lower ranges, is unknown and needs further studies and clinical validations.

## New knowledge gained

We demonstrate in a large patient population that CACS calculated with low-dose CT scan correlates well with CACS assessed with standard protocols, but there is a tendency toward an underestimation which may possibly limit its prognostic value.

## Conclusion

In a large consecutive patient population, CACS calculated with low-dose CCT scan and those assessed with standard protocol correlated well regarding numeric CACS and risk classes. However, there was a systematic underestimation of CACS with low-dose protocols, causing a significantly higher proportion of patients without detectable coronary calcifications. This may impact the prognostic value of CACS and the established “power of zero” may no longer be warranted if the calcium score is assessed using CT with low tube voltages.

## Supplementary Information

Below is the link to the electronic supplementary material.

Supplementary file1 (MP3 2292 kb)

Supplementary file2 (PPTX 394 kb)

## Abbreviations

AC: Attenuation correction
CACS: Coronary artery calcium score
CAD: Coronary artery disease
AC: Attenuation correction
CCT: Cardiac computer tomography
LAD: Left anterior descending artery
RCA: Right coronary artery
LCX: Left circumflex artery
MPS: Myocardial perfusion SPECT-CT
SPECT: Single photon emission computed tomography
SRS/SSS/SDS: Summed rest/stress/difference score
