# Supplementary material for: Low-dose coronary artery calcium scoring compared to the standard protocol
Source: J Nucl Cardiol. 2022 Oct 26;30(3):1191–8. doi: 10.1007/s12350-022-03120-3 (PMC10261226; doi:10.1007/s12350-022-03120-3)
Supplement: Supplementary file 2 — Supplementary file2 (PPTX 394 kb) [file 12350_2022_3120_MOESM2_ESM.pptx]

## Slide 1
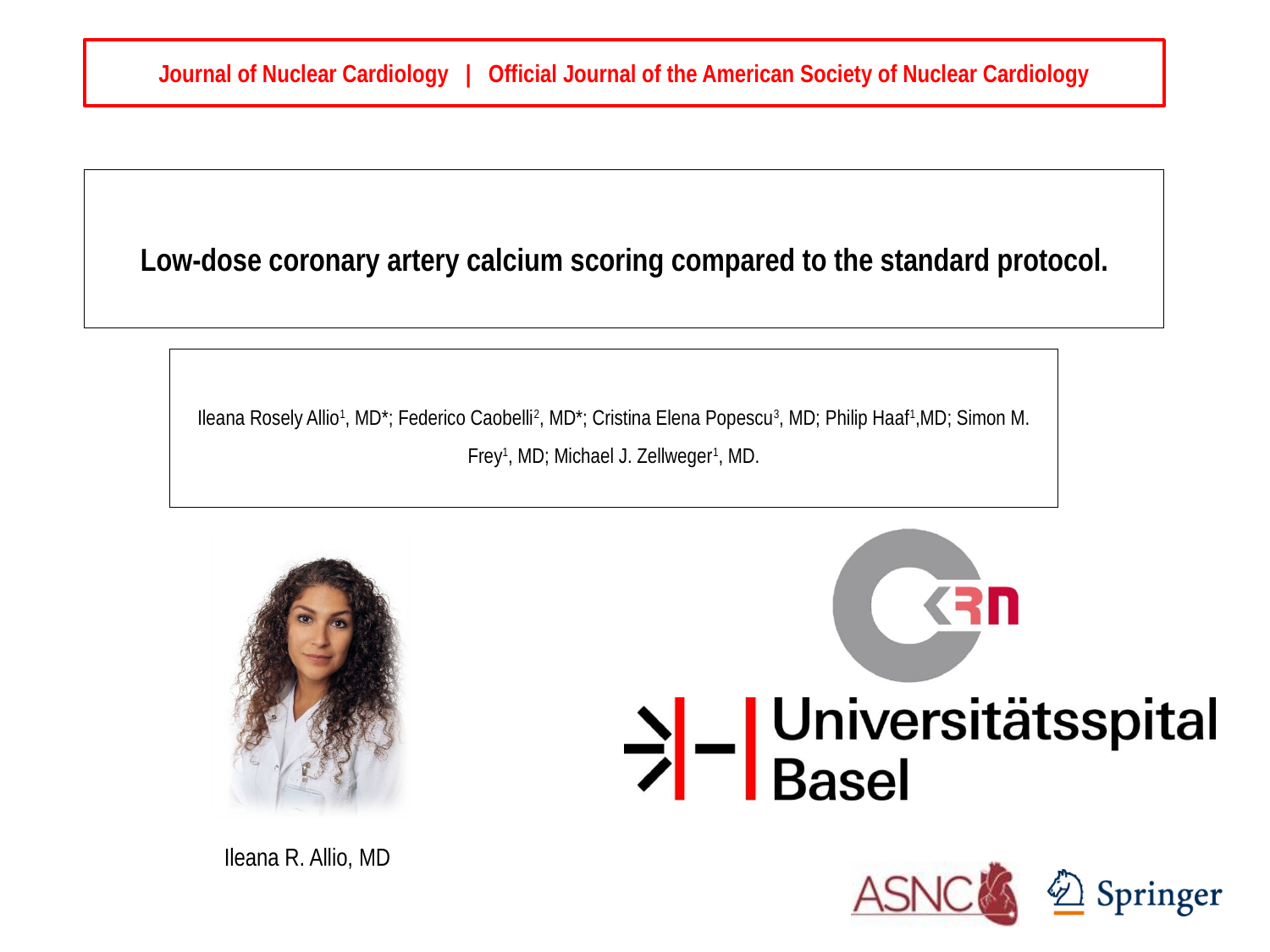

Journal of Nuclear Cardiology | Official Journal of the American Society of Nuclear Cardiology
# Low-dose coronary artery calcium scoring compared to the standard protocol.
Ileana Rosely Allio1, MD*; Federico Caobelli2, MD*; Cristina Elena Popescu3, MD; Philip Haaf1,MD; Simon M. Frey1, MD; Michael J. Zellweger1, MD.
Ileana R. Allio, MD

## Slide 2
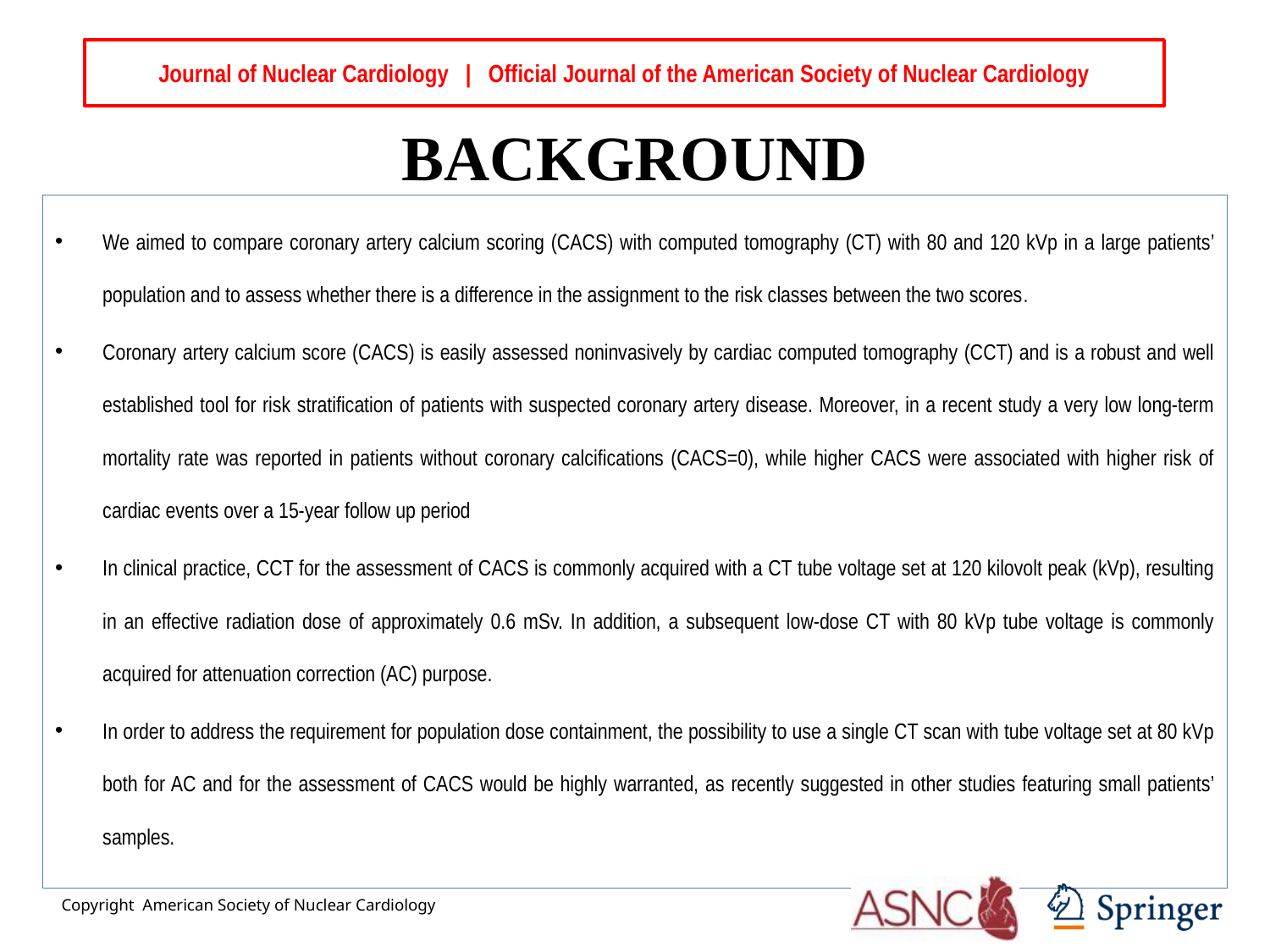

Journal of Nuclear Cardiology | Official Journal of the American Society of Nuclear Cardiology
# BACKGROUND
We aimed to compare coronary artery calcium scoring (CACS) with computed tomography (CT) with 80 and 120 kVp in a large patients’ population and to assess whether there is a difference in the assignment to the risk classes between the two scores.
Coronary artery calcium score (CACS) is easily assessed noninvasively by cardiac computed tomography (CCT) and is a robust and well established tool for risk stratification of patients with suspected coronary artery disease. Moreover, in a recent study a very low long-term mortality rate was reported in patients without coronary calcifications (CACS=0), while higher CACS were associated with higher risk of cardiac events over a 15-year follow up period
In clinical practice, CCT for the assessment of CACS is commonly acquired with a CT tube voltage set at 120 kilovolt peak (kVp), resulting in an effective radiation dose of approximately 0.6 mSv. In addition, a subsequent low-dose CT with 80 kVp tube voltage is commonly acquired for attenuation correction (AC) purpose.
In order to address the requirement for population dose containment, the possibility to use a single CT scan with tube voltage set at 80 kVp both for AC and for the assessment of CACS would be highly warranted, as recently suggested in other studies featuring small patients’ samples.
Copyright American Society of Nuclear Cardiology

## Slide 3
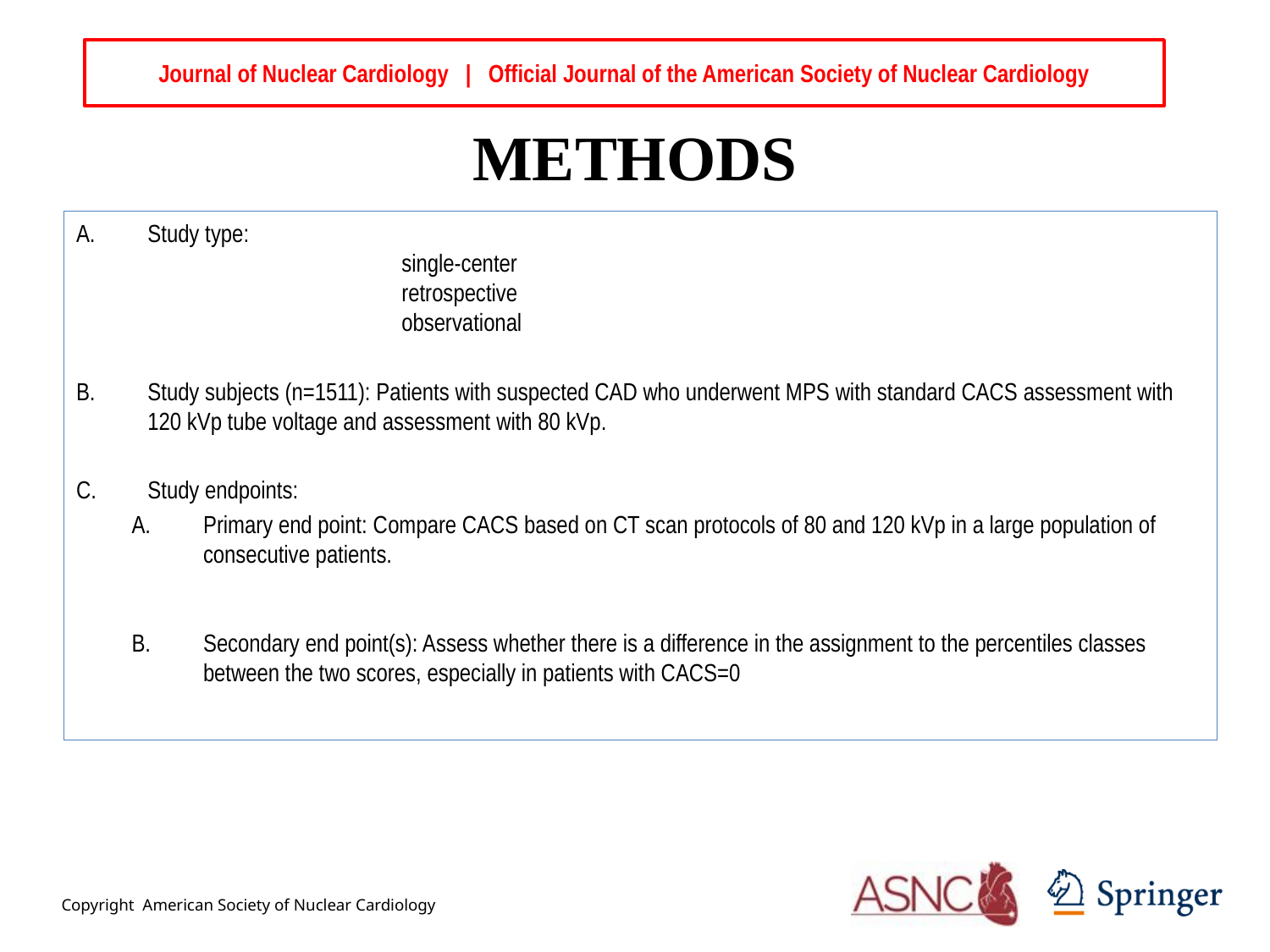

Journal of Nuclear Cardiology | Official Journal of the American Society of Nuclear Cardiology
# METHODS
Study type:
		single-center
		retrospective
		observational
Study subjects (n=1511): Patients with suspected CAD who underwent MPS with standard CACS assessment with 120 kVp tube voltage and assessment with 80 kVp.
Study endpoints:
Primary end point: Compare CACS based on CT scan protocols of 80 and 120 kVp in a large population of consecutive patients.
Secondary end point(s): Assess whether there is a difference in the assignment to the percentiles classes between the two scores, especially in patients with CACS=0
Copyright American Society of Nuclear Cardiology

## Slide 4
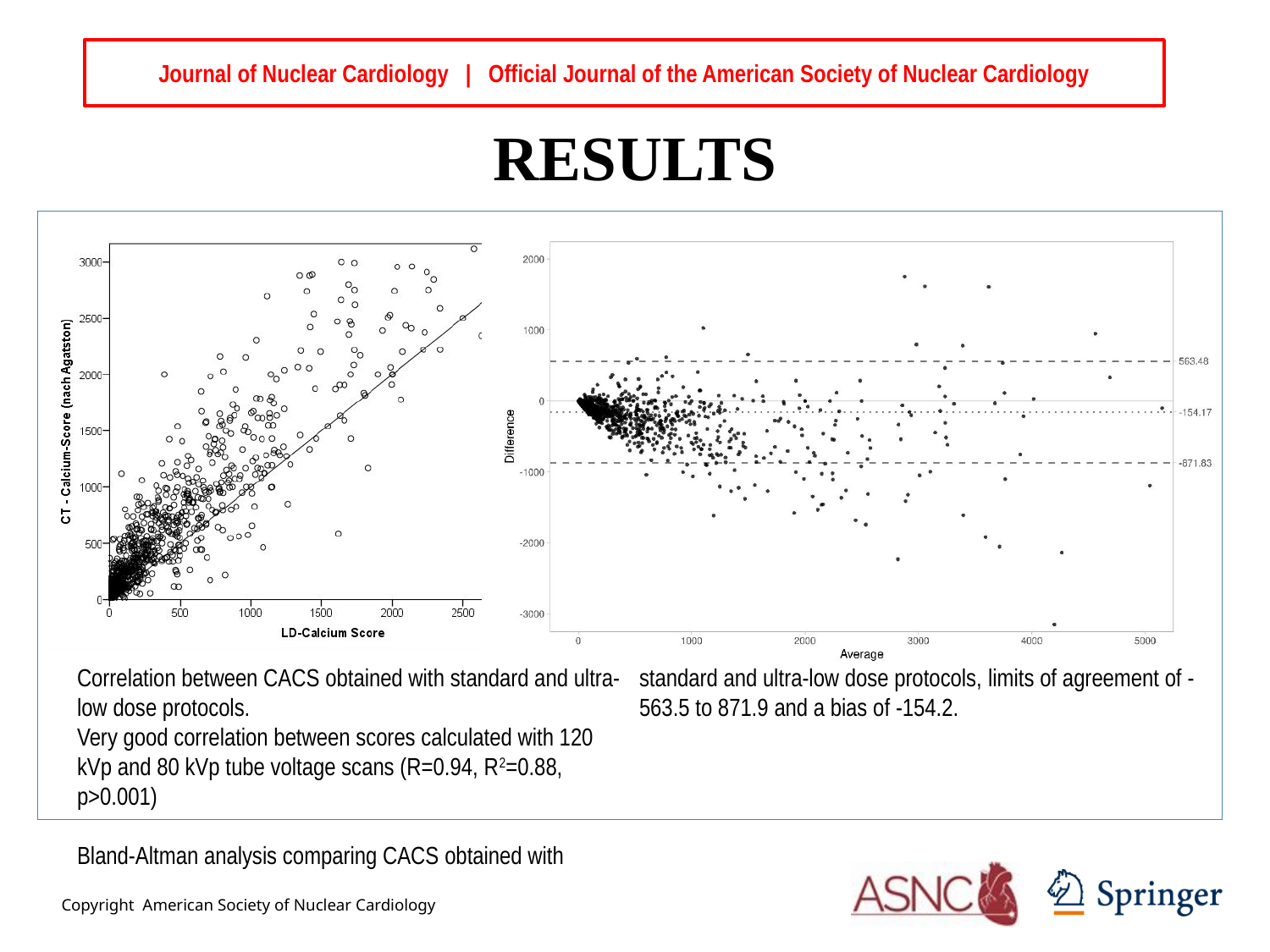

Journal of Nuclear Cardiology | Official Journal of the American Society of Nuclear Cardiology
# RESULTS
Correlation between CACS obtained with standard and ultra-low dose protocols.
Very good correlation between scores calculated with 120
kVp and 80 kVp tube voltage scans (R=0.94, R2=0.88, p>0.001)
Bland-Altman analysis comparing CACS obtained with standard and ultra-low dose protocols, limits of agreement of -563.5 to 871.9 and a bias of -154.2.
Copyright American Society of Nuclear Cardiology

## Slide 5
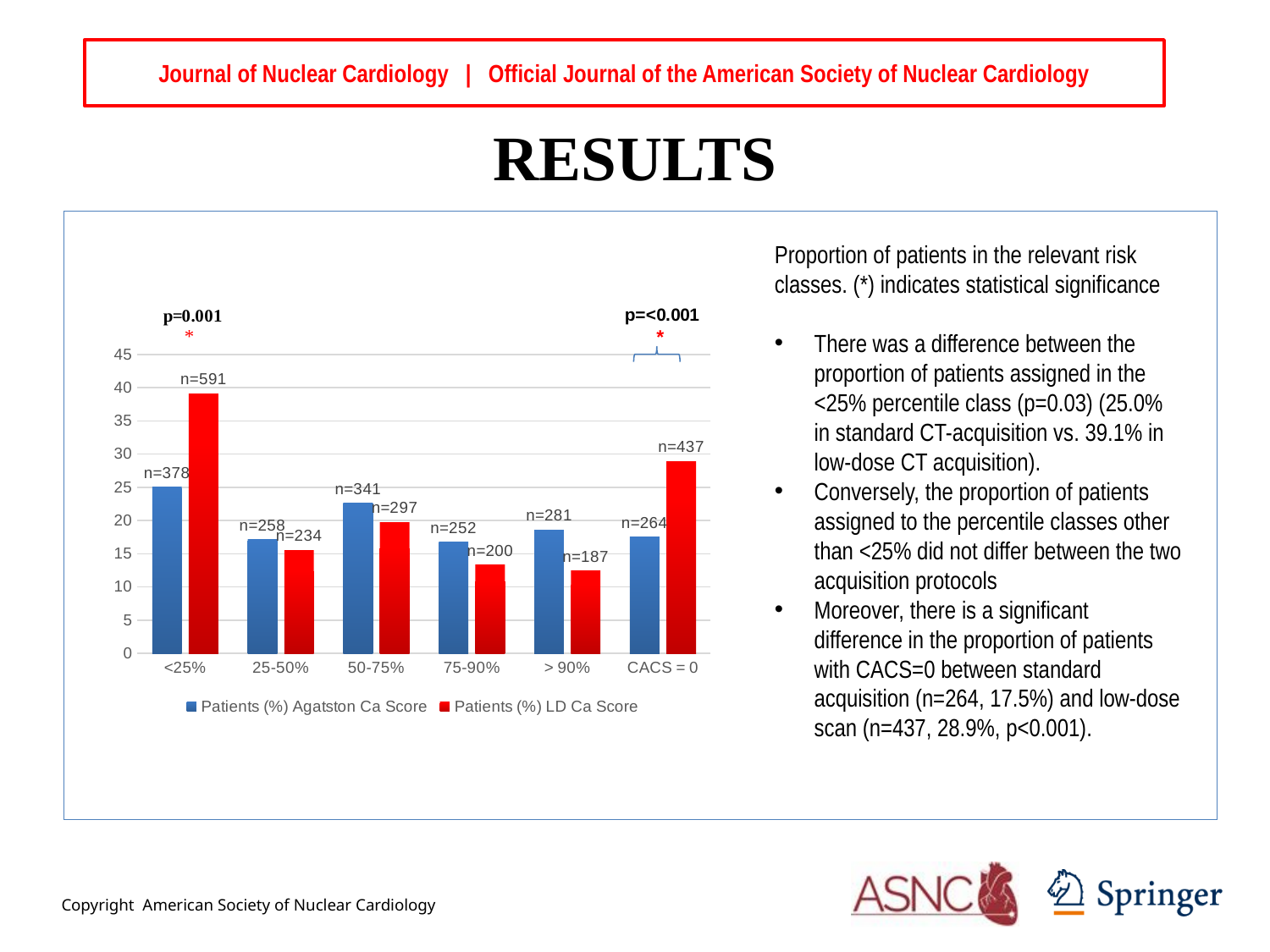

Journal of Nuclear Cardiology | Official Journal of the American Society of Nuclear Cardiology
# RESULTS
Proportion of patients in the relevant risk classes. (*) indicates statistical significance
There was a difference between the proportion of patients assigned in the <25% percentile class (p=0.03) (25.0% in standard CT-acquisition vs. 39.1% in low-dose CT acquisition).
Conversely, the proportion of patients assigned to the percentile classes other than <25% did not differ between the two acquisition protocols
Moreover, there is a significant difference in the proportion of patients with CACS=0 between standard acquisition (n=264, 17.5%) and low-dose scan (n=437, 28.9%, p<0.001).
### Chart
| Category | Patients (%) Agatston Ca Score | Patients (%) LD Ca Score |
|---|---|---|
| <25% | 25.0 | 39.1 |
| 25-50% | 17.1 | 15.5 |
| 50-75% | 22.6 | 19.7 |
| 75-90% | 16.7 | 13.3 |
| > 90% | 18.6 | 12.4 |
| CACS = 0 | 17.5 | 28.9 |
Copyright American Society of Nuclear Cardiology

## Slide 6
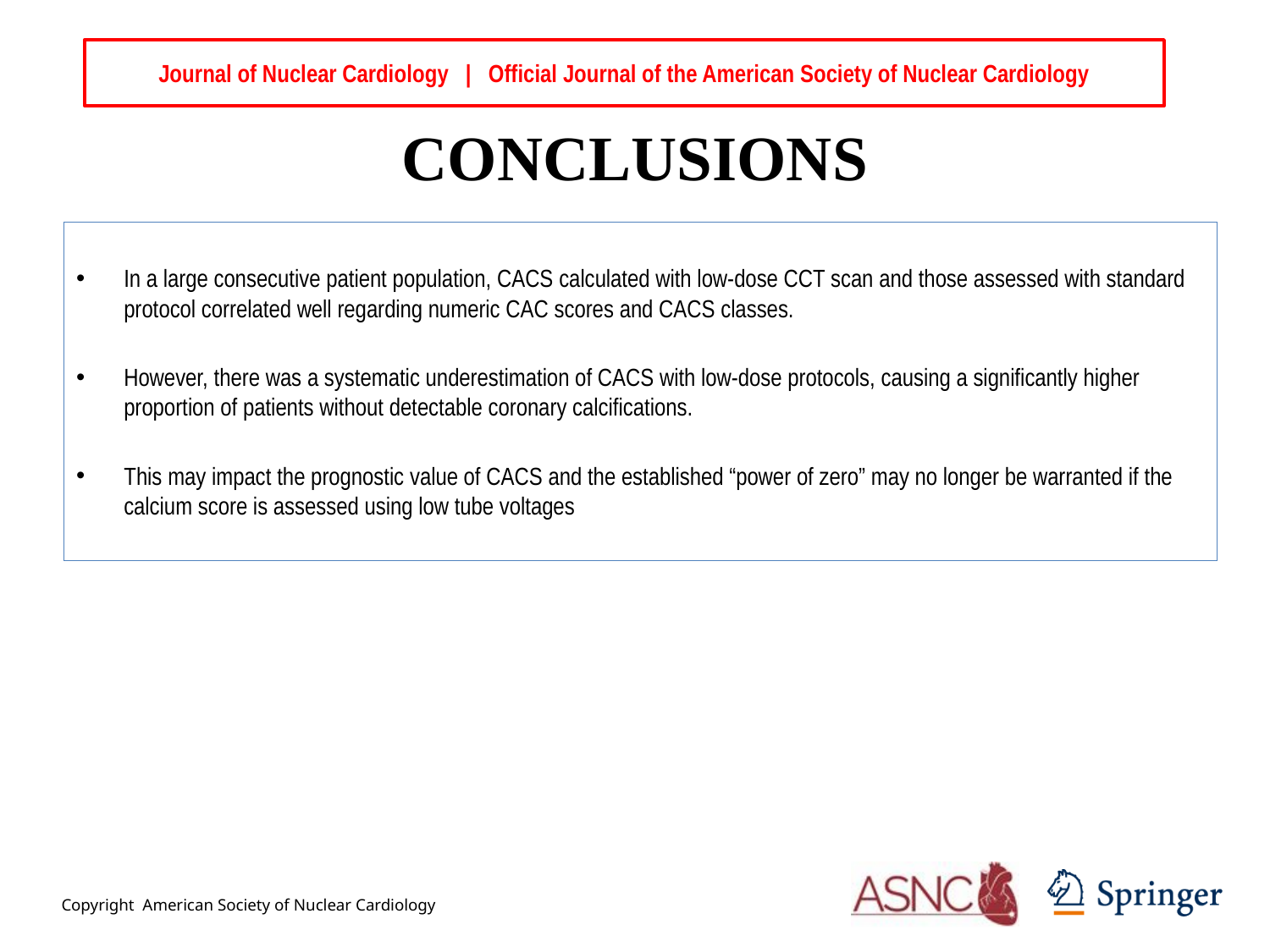

Journal of Nuclear Cardiology | Official Journal of the American Society of Nuclear Cardiology
# CONCLUSIONS
In a large consecutive patient population, CACS calculated with low-dose CCT scan and those assessed with standard protocol correlated well regarding numeric CAC scores and CACS classes.
However, there was a systematic underestimation of CACS with low-dose protocols, causing a significantly higher proportion of patients without detectable coronary calcifications.
This may impact the prognostic value of CACS and the established “power of zero” may no longer be warranted if the calcium score is assessed using low tube voltages
Copyright American Society of Nuclear Cardiology
